# An Injectable Oil-Based Depot Formulation of *N*-Acyloxymethyl Prodrug of Ropivacaine for Long-Acting Local Analgesia: Formulation Development and In Vitro/In Vivo Evaluation

**DOI:** 10.3390/pharmaceutics17010037

**Published:** 2024-12-30

**Authors:** Xiaowei Liu, Ruihan Zhao, Peijie Xu, Jianqiang Qian, Peiyan Zhang, Xudong Xie, Yong Ling, Qimin Ge, Yong Chen

**Affiliations:** 1 School of Pharmacy, Nantong University, 9 Seyuan Road, Nantong 226019, China; 2119320015@stmail.ntu.edu.cn (X.L.);; 2 School of Pharmacy, China Pharmaceutical University, 639 Longmian Avenue, Nanjing 211198, China; zrh016940@outlook.com; 3 Jiangsu Provincial Institute of Materia Medica, 26 Majia Street, Nanjing 211816, China; 4 Department of Pharmacy, The First People’s Hospital of Yancheng, 66 Renmin South Road, Yancheng 224006, China

**Keywords:** ropivacaine, prodrug, oil depot, long-acting injectables, sustained release, local analgesia, postoperative pain

## Abstract

**Objectives:** The development of novel long-acting injectables for local anesthetics is necessary to effectively manage the acute postoperative pain. The aim of this study was to prepare an injectable oil-based formulation of ropivacaine (ROP) prodrug (ropivacaine stearoxil, ROP-ST) and to investigate the pharmacokinetics and pharmacodynamics after injectable administration. **Methods:** A novel *N*-acyloxymethyl prodrug of ROP, i.e., ROP-ST, was synthesized and its physicochemical properties such as log P, solubility and stability characterized. A soybean oil-based depot of ROP-ST was prepared, and the in-vitro release of ROP-ST was evaluated using an “inverted-cup” method. Pharmacokinetic profiles and tissue retention properties were investigated after intramuscular administration of the formulation in rats. The analgesic efficacy was assessed via a von Frey monofilaments test by measuring the paw withdrawal thresholds. **Results:** The structure of ROP-ST was ascertained with clear ^1^H NMR assignment and accurate mass-to-charge ratio. The high Log P value of ROP-ST (9.16) demonstrated extremely low aqueous solubility, but the prodrug is biolabile when in contact with plasma or liver esterase. Intramuscular injection of ROP-ST oil solution in rats provided a significantly higher mean residence time without a very clear plasma peak of ROP. In a postoperative pain model of rats, the injection of ROP-ST oil solution into the vicinity of the sciatic nerve in the right ankle effectively controlled the postoperative pain for at least 72 h. **Conclusions:** The injectable oil-based depot formulation of *N*-acyloxymethyl prodrug of ROP may provide a new opportunity of long-acting local analgesia for postoperative pain.

## 1. Introduction

Acute postoperative pain occurs after surgery and usually lasts for 3~7 days [1]. Inadequate treatment of this type of pain usually results in increased morbidity, delayed recovery, and reduced patient satisfaction [2]. Local anesthetics (LASs) can block the production and conduction of local sensory nerve impulses when acting on local nerve endings or nerve trunks at appropriate concentrations. LASs are widely used in clinical anesthesia and postoperative pain management because they are injected locally, leading to reductions of systemic adverse reactions compared with oral opioid analgesics or non-steroidal anti-inflammatory drugs (NSAIDs). After a single administration of LASs, the duration of action normally lasts for ˂6 h. Therefore, multiple injections of LASs are usually required for the effective management of postoperative pain in clinical scenarios.

To reduce the frequency of LAS injections, long-acting injectables for LASs have been developed. For example, bupivacaine liposome injectable suspension (Exparel^®^) is administered into the surgical site to produce postsurgical analgesia with superior clinical benefit: the sensory block was nearly 4 times longer in patients who received the liposome bupivacaine mixture compared with patients who received bupivacaine hydrochloride alone [3]. Zynrelef^®^, developed by Heron Therapeutics, is a mixture of bupivacaine and a small dose of meloxicam in a fixed ratio, loaded into a sustained-release excipient polyorthoesters, and injected into the surgical incision site. Meloxicam can reduce the inflammatory response at the surgical site and restore the pH value of the surgical incision site to normal, while analgesia is maintained by bupivacaine [4]. Posimir^®^ is a solution system using a biodegradable polymer sucrose acetate isobutyrate (SAIB) to incorporate bupivacaine, which forms a gel after injection at the sutured wound site, delaying the release of bupivacaine [5]. Many novel delivery systems aiming to prolong the duration of LASs, such as injectable particles in situ gels triggered formulations and hybrid systems, were investigated in the preclinical stage [5,6,7,8]. However, some of these complex formulations suffer from complicated manufacturing processes, high expense, or even some intrinsic drawbacks, e.g., liposomes may have instability issues.

Prodrug strategy is a powerful tool to modulate the release profiles of drugs in vivo. Many clinically successful sustained-release products for long-acting therapy were developed by using this strategy. For example, paliperidone palmitate once-monthly injection (Invega Sustenna^®^) was developed by Janssen Pharmaceuticals to improve medication adherence for the maintenance treatment of schizophrenia. Nalbuphine sebacate (Naldebain^®^) was developed by Lumosa Therapeutics for the relief of moderate/severe postoperative pain for 7 days without the side effects associated with opioids. It was also reported in our group previously that the effective management of acute postoperative pain for a longer duration can be achieved in a rat model using intravenous emulsions of novel ketorolac prodrugs [9]. Recently, a thermoresponsive *N*-(2-hydroxypropyl) methacrylamide copolymer-based hydromorphone prodrug was developed to achieve sustained local analgesia without apparent adverse effects [10].

The use of prodrug strategies to prolong the half-life of drugs has several advantages. The mechanism of sustained release of drug is very clear: slow drug release can be achieved by the dissolution of lipophilic prodrug from the formulation and the cleavage of a biolabile linker—mostly through hydrolysis of ester bond by esterase at the injection site. For example, fluphenazine decanoate and haloperidol decanoate long-acting agents are synthesized such that the parent drugs are esterified to fatty acid chains that allow the agents to be dissolved in sesame oil. Administration of the oily solution into deep muscle tissue as a “depot” results in a pool of medication that is subsequently hydrolyzed in situ through an inflammatory response, which releases the parent drug into the systemic circulation [11]. Similarly, after intramuscular (IM) injection of paliperidone palmitate, which is manufactured based on nanocrystal technology, the suspended palmitate conjugates are firstly hydrolyzed to the active drug, which then dissolves slowly into the systemic circulation [12]. Due to the very low solubility of paliperidone palmitate, slow dissolution occurs at the injection site [13]. It should be noted that the development of a prodrug may be considered a new chemical entity, which could increase the investment during R&D. However, in some cases, synthesizing and formulating such prodrugs in a full-scale commercial production level could be simpler than manufacturing a complex formulation, and the storage conditions of these formulations could be more eco-friendly. To the best of our knowledge, previous publications on the use of prodrug strategy to prolong or control the release of LASs are very limited [14,15].

**Figure 1:**
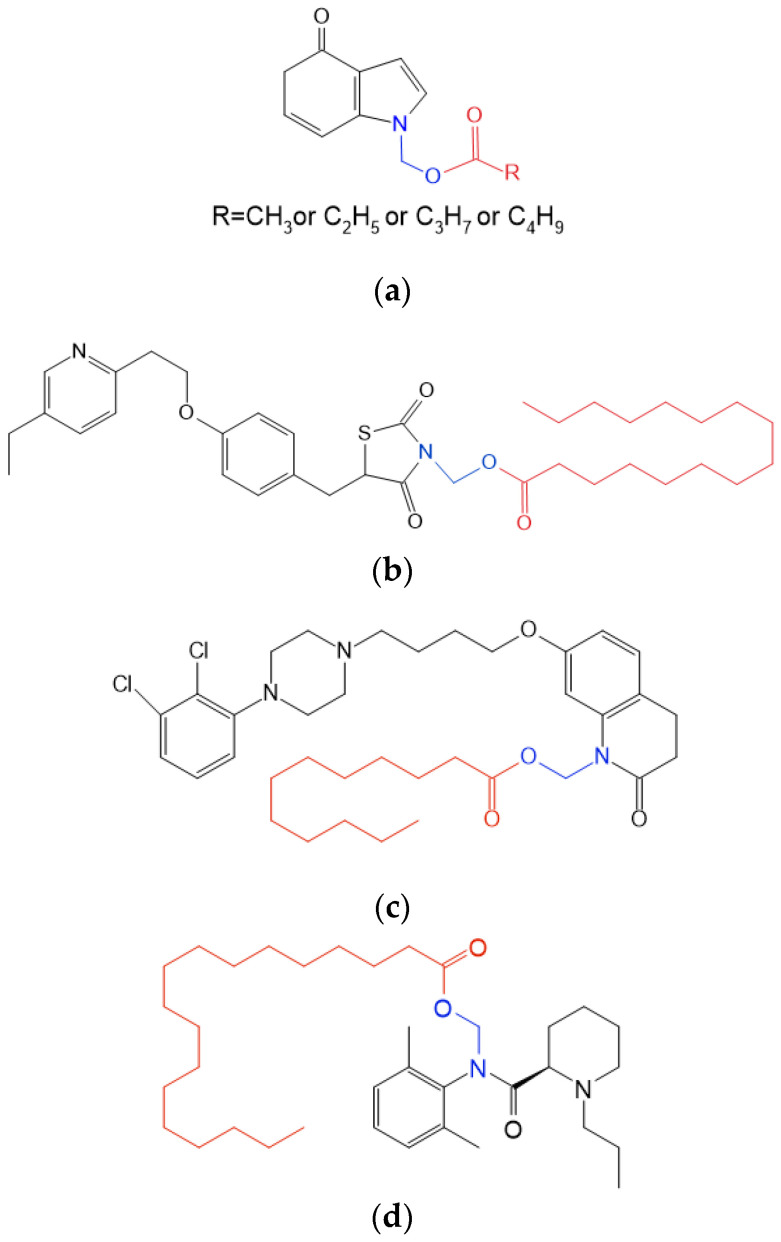
The structures of *N*-acyloxymethyl derivatives of allopurinol (**a**); a palmitoyloxymethyl derivative of pioglitazone (**b**); aripiprazole lauroxil (**c**); and ROP-ST (**d**). The structures in black are the parent drugs, the structures in red are fatty acid moieties, and the structures in blue are the *N*-methyleneoxy linkers.

Ropivacaine (ROP) is an amide-type LAS developed by AstraZeneca. ROP is known for its longer-lasting analgesic effects compared to the ester-type LAS, such as procaine. A variety of injectable formulations of ROP aiming to achieve long-acting analgesia were developed, including microspheres [16], liposomes [7], in situ hydrogel [17,18], nanoparticles [19] and nanocrystals [20], etc. However, the prodrug of ROP has not been reported. ROP lacks easily modified functional groups such as hydroxyl and carboxyl groups, which renders the chemical modification by simple esterification difficult. *N*-acyloxyalkylation of *NH*-acidic compounds is a prodrug approach for tertiary or some *N*-heterocyclic amines and secondary amides and has the potential to modify the properties of the parent drug for specific uses [21,22]. For example, a study on *N*-acyloxymethyl prodrugs of allopurinol (Allop, Figure 1a) showed that the prodrugs were turned into parent drugs slowly in vivo, by which their side effects were reduced [23]. A palmitoyloxymethyl derivative of pioglitazone (Figure 1b) also presented commendable potential as a once-monthly injectable medication to treat diabetes [24]. Aripiprazole lauroxil, an *N*-acyloxymethyl prodrug of aripiprazole (Figure 1c), was developed for long-term treatment of various psychiatric disorders (Aristada^®^). Theoretically, *N*-acyloxyalkyl derivates of *NH*-acid compounds undergo a two-step bioconversion into the parent *NH*-acidic drug through an unstable *N*-hydroxyalkyl intermediate [21]. Inspired by the above-mentioned examples, we assumed that a lipophilic prodrug of ROP, i.e., ropivacaine stearoxil (ROP-ST, Figure 1d), formed by *N*-acyloxyalkylation of ROP with stearic acid moiety, could be solubilized in injectable soybean oil and release ROP slowly in situ after enzymatic hydrolysis, thereby achieving long-acting local analgesia.

The specific aims of this study were (i) to synthesize, purify and characterize the ROP-ST prodrug, (ii) to determine the physicochemical properties of ROP-ST, (iii) to prepare and evaluate an oil depot formulation of ROP-ST, (iv) to compare pharmacokinetic profiles of ROP after IM administration of an aqueous solution of ROP hydrochloride, an oil solution of ROP (free base), and an oil solution of ROP-ST in rats and (v) to evaluate the analgesic effects of these formulations using postoperative pain model in rats.

## 2. Materials and Methods

### 2.1. Materials

The ropivacaine hydrochloride powder (ROP·HCl, batch No. K901856) was purchased from Dibai Biotech (Shanghai, China); aripiprazole lauroxil (APL, batch No. ARI001001) was obtained from Huixin Medical Technology (Hefei, China); porcine liver esterase (15 U/mg, batch No. C14299830) was bought from McLean Biochem (Shanghai, China); injectable soybean oil (batch No. p2363081, purity ≥ 99%) was bought from Titian Scientific (Shanghai, China); HPLC-grade acetonitrile and methanol were supplied by Merck KGaA (Darmstadt, Germany). All aqueous solutions were prepared using ultrapure water (resistivity > 18 MΩ.cm). The ultrapure water was obtained from a Direct-Q^®^ 5 UV Water Purification System (Millipore S.A.S.; Molsheim, France). All other chemicals were at least of analytical grade and used directly without purification.

### 2.2. Animals

Male Sprague–Dawley (SD) rats (B.W. 220 ± 20 g) were supplied by the Laboratory Animal Center of Nantong University. The in vivo studies adhered to the ethical guidelines and regulations approved by the Animal Experimentation Ethics Committee at Nantong University (protocol proof: S20240617-005). The ethical guideline follows the “Laboratory animal—Guideline for ethical review of animal welfare”. All animals were in a 12 h dark–light cycle animal facility with controlled temperature and humidity and had free access to regular chow and water except for the test sessions.

### 2.3. Synthesis and Characterization of ROP-ST

**Figure 2:**
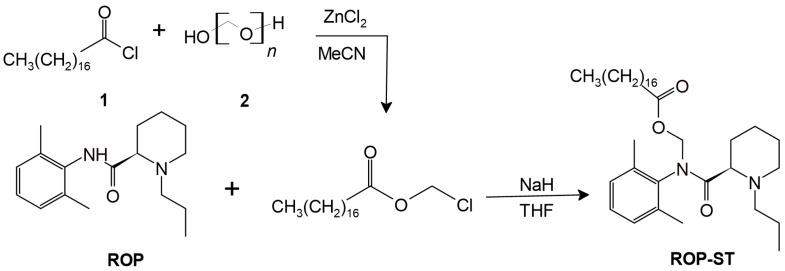
The synthesis of ROP-ST. 1. Stearoyl chloride; 2. polyformaldehyde.

The synthetic route is shown in Figure 2. ROP·HCl (1 g, 3.24 mmol) was dissolved in a flask containing 20 mL of pure water, to which 2 g of Na_2_CO_3_ was added, and the mixture was stirred for 1 h. Subsequently, 40 mL of ethyl acetate was added, and the mixture was stirred for 0.5 h. The organic phase was isolated and dried by anhydrous Na_2_SO_4_. The solvent was removed by rotary evaporation to obtain the free base of ROP·HCl, i.e., ROP.

In the next step, ROP was subjected to a *N*-acyloxymethylation modification. Briefly, to 50 mL of anhydrous acetonitrile, polyformaldehyde (0.18 g; 6 mmol, calculated by formaldehyde), and anhydrous zinc chloride (0.82 g, 6 mmol) were added. The mixture was stirred under N_2_ protection on an ice bath, to which stearoyl chloride (1.81 g, 6 mmol) was added dropwise. The solution was stirred for 1 h on the ice bath, and then warmed up to 50 °C. The reaction ran overnight, followed by removing the solid reagent by filtration. The filtrate was concentrated under reduced pressure to obtain *N*-acyloxymethyl chloride, which was used in the subsequent reaction directly without further purification. NaH (0.48 g, 20 mmol) was dispersed into 30 mL of ice-cold THF, to which ROP (0.82 g, 3 mmol, dissolved in THF) was added. The mixture was stirred on an ice bath, to which *N*-acyloxymethyl chloride (dissolved in THF) was then added dropwise, and the reaction lasted for another 2 h. After completion of the reaction, the mixture was concentrated by rotary evaporator, poured into water, extracted with ethyl acetate, and dried over anhydrous Na_2_SO_4_. The solvent was removed under reduced pressure, and the residue was purified by column chromatography to yield a colorless oily product, i.e., ROP-ST. The purity was >98% based on the area normalization method using an HPLC-UV. The structure of ROP-ST was ascertained by ^1^H NMR (AVANCE III HD 400 NMR Spectrometer, Bruker Corporation, Billerica, United States) and high-resolution mass spectrometry. The spectrum of ^1^H NMR and high-resolution mass spectrometry (API QSTAR® Pulsar ESI-Qtof Mass Spectrometer; Applied Biosystems, Waltham, United States) can be found in the Supplemental Materials.

ROP-ST: ^1^H NMR (400 MHz, CDCl_3_) δ: 0.88 (t, *J* = 3.7 Hz, 6 H, 2CH_3_), 1.69–1.42 (m, 16 H, 8CH_2_), 2.01 (m, 5 H, CH_3_, CH_2_), 2.19 (t, *J* = 6.0 Hz, 6 H, 3CH_2_), 2.34 (m, 8 H, 4CH_2_), 2.63 (m, 2 H, CH_2_), 3.24~3.14 (m, 2 H), 3.46 (dd, *J* = 9.6, 3.3 Hz, 1 H), 5.43 (m, 1 H, CH), 5.80 (dd, *J* = 19.0, 10.4 Hz, 2 H, CH_2_), 7.14 (m, 2 H, 2ArH), 7.19 (dd, *J* = 8.5, 6.4 Hz, 1 H, ArH). HRESI-MS (C_36_H_62_N_2_O_3_ + H): Calc. 571.4839; Exp. 571.4826.

### 2.4. Solubility and Partition Coefficient

The solubility of ROP-ST and ROP was determined by adding an excessive amount of each compound into 5 mL of pure water, PBS buffers (pH 7.4), and soybean oil, respectively, followed by agitating in a water bath shaker (37 °C) for 72 h. After centrifugation at 12,000 r/min for 10 min (TGL-16C centrifuge; Anting Scientific Instrument Company, Shanghai, China), the supernatant was withdrawn, and the solution was filtered through a syringe filter (pore size: 0.45 μm). After appropriate dilution with acetonitrile, the concentrations of ROP-ST and ROP were analyzed using HPLC−UV. The experiments were performed in triplicates.

Approximately 1 mg of ROP-ST and ROP-free base were dissolved in a bi-phase mixture of water-saturated *n*-octanol solution and *n*-octanol-saturated water (5 mL:5 mL), respectively. After equilibration in a water bath shaker (37 °C) for 24 h, the two phases were sampled separately and diluted with acetonitrile. The concentrations of ROP-ST and ROP were determined using HPLC−UV. The Log P value of each compound was calculated accordingly. The experiments were performed in triplicates. In addition, the Log P values of ROP and ROP-ST were predicted using an on-line Molinspiration software (https://www.molinspiration.com/, accessed on 31 March 2023).

Quantification of ROP-ST was performed using an HPLC−UV system consisting of an LC-10AT VP pump, an SPD-10A VP UV–vis detector, a CTO-10A column oven, a SIL-10AF autosampler, and an SCL-10A VP controller (Shimadzu Corporation; Kyoto, Japan). Data were collected and processed using LC-solution software. Isocratic elution was performed on a Diamonsil C18 column (150 mm × 4.6 mm I.D., 5 µm), and an EasyGuard C18 guard column (10 × 4.0 mm I.D., 5 µm) was mounted upstream from the analytical column (Dikma Technologies; Beijing, China). The column temperature was kept at 35 °C, and the UV absorbance wavelength was set at 215 nm. The mobile phase, comprising 88% (*v*/*v*) acetonitrile and 12% (*v*/*v*) water with 0.1% (*v*/*v*) trifluoroacetic acid, was used after filtering through a filter membrane (pore size: 0.45 μm) and degassing within an ultrasonic water bath (KQ-500VDE Ultrasonic Cleaner; Kunshan, China) for 40 min. The flow rate was maintained at 1.0 mL/min, and the injection volume was 20 µL. The calibration curve was linear in the range of 1–200 μM, and the correlation coefficient was >0.9998. The method showed good precision and accuracy for both intra- and inter-day analyses. The lower limit of quantification (LOQ) of ROP-ST was 1 μM (equal to 0.57 μg/mL). The validation of the HPLC method can be found in Supplemental Materials.

Quantification of ROP was performed using the same equipment and columns as described above, and the quantification method was adapted from the Chinese Pharmacopoeia (2020 edition). Briefly, the column temperature was kept at 35 °C, and the UV absorbance wavelength was set at 240 nm. The mobile phase comprised 50% (*v*/*v*) acetonitrile and 50% (*v*/*v*) phosphate buffer. The phosphate buffer was prepared by mixing 1.3 mL of sodium dihydrogen phosphate (1 mol/L) with 32.5 mL of disodium hydrogen phosphate (0.5 mol/L) and diluting to 1 L by water, and then the pH value was adjusted to 8.0. The flow rate was maintained at 1.0 mL/min, and the injection volume was 20 µL. The validation of the quantification method can be found in the Supplementary Materials.

### 2.5. Biological Stability

#### 2.5.1. Plasma Stability Study

Healthy male Sprague–Dawley rats (SD rats, 200 ± 20 g) were provided by the Animal Experiment Center of Nantong University (protocol code of the ethical proof: S20240617-005). Blood samples were collected from the orbital cavity and centrifuged at 10,000 r/min for 10 min (TGL-16C centrifuge; Anting Scientific Instrument Company, Shanghai, China) to obtain blank plasma. The blank plasma was mixed with saline to obtain plasma solutions of 10%, 20%, and 40% (*v*/*v*) plasma, respectively [9]. A half milliliter of saline, or 10%, 20%, and 40% plasma solution, was added to an EP tube, respectively, followed by adding 50 μL of methanolic solution of ROP-ST (concentration: 1100 μM) to each tube. The tubes were sealed and placed in a water bath shaker (37 °C). Three tubes were taken at each predetermined time point (0, 10, 20, 30, 40, 50, 60, 70, 80, and 90 min, respectively), rapidly frozen in liquid nitrogen to terminate the enzymatic reaction, and then stored at −80 °C. The samples were lyophilized to obtain dry residues. Before analysis, the residues were dissolved in methanol and subjected to ultrasonic treatment for 30 s. The resulting solution was filtered through a filter membrane (pore size: 0.45 μm), and the remaining levels of ROP-ST in subsequent filtrates were quantified by HPLC−UV. The hydrolysis rate constants (*k*_obs_) of the prodrug were calculated by plotting the natural logarithm of the remaining percentage against time. The corresponding half-life (*t*_1/2_) was then calculated (*t*_1/2_ = ln2/*k*_obs_) [25]. The experiment was conducted in triplicates.

#### 2.5.2. Liver Esterase Stability Study

Porcine liver esterase solutions were prepared by dissolving 4, 8, and 16 mg of porcine liver esterase powder (15 U/mg) into 12 mL of phosphate buffer (pH 8.0) to obtain porcine liver esterase solutions with the concentrations of 5, 10 and 20 U/mL, respectively [24]. A half milliliter of blank buffer solution (pH 8 without enzyme) or porcine liver esterase solutions with the concentration of 5, 10, or 20 U/mL, respectively, was added to an EP tube, followed by adding 50 μL of methanolic solution of ROP-ST (concentration: 1100 μM) to each tube. The tubes were sealed and placed in a water bath shaker (25 °C) according to product instructions and a previous report [26]. Three tubes were taken at each predetermined time point (0, 30, 45, 60, 75, 90, 105, and 120 min, respectively), rapidly frozen in liquid nitrogen to terminate the enzymatic reaction, and then stored at −80 °C. The subsequent treatments of the samples complied with the procedures described in “Section 2.5.1.”. The experiment was conducted in triplicates.

### 2.6. Preparation and In Vitro Evaluation of ROP-ST Oil Solution

#### 2.6.1. Formulation Preparation

To prepare the ROP-ST oil solution for an injectable depot, 30 mL of soybean oil was placed into a transparent glass bottle, to which 469 mg of ROP-ST was mixed and dissolved. The final concentration of ROP-ST was 15.6 mg/mL, which equaled 7.5 mg/mL (27.37 mM) of ROP. It should be noted that the marketed ropivacaine solution for injection is also 7.5 mg/mL. The formulation was dispensed into sterile glass vials (1 mL for each vial), filled with nitrogen, sealed, sterilized at 121 °C for 15 min, and stored at room temperature.

#### 2.6.2. Assay

A total of 100 mL of acetonitrile was used to dilute 500 μL of ROP-ST soybean oil solution. The samples were sonicated for 30 min, shaken for 5 min, and filtered through a filter membrane (pore size: 0.45 μm). The subsequent filtrate was subjected to quantification of ROP-ST by HPLC-UV. The content (%) of ROP-ST was calculated by dividing the measured concentration by the theoretical concentration. The procedure was performed in triplicates.

#### 2.6.3. Storage Stability

The stability of ROP-ST in its oil solution was tested by placing the formulation at room temperature for 6 months or at a high temperature (50 °C) for 3 months. The content of ROP-ST was assayed using the HPLC method described in “Section 2.4”. The tests were performed in triplicates.

#### 2.6.4. In Vitro Drug Release

**Figure 3:**
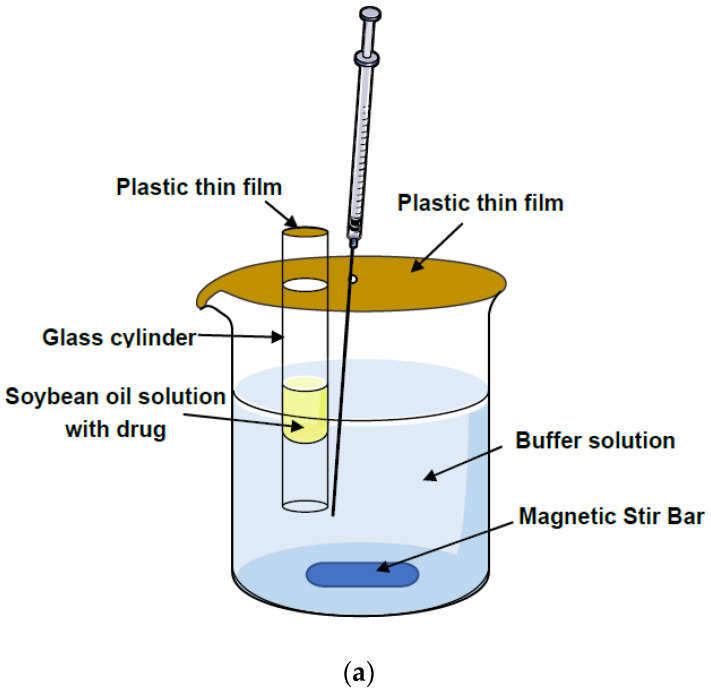

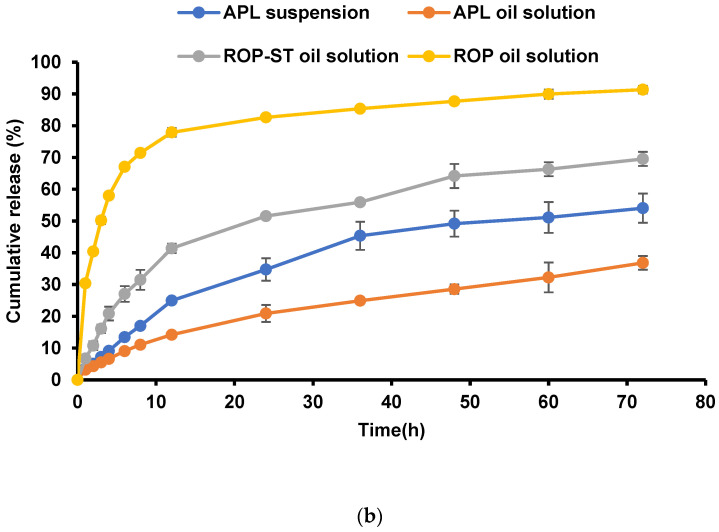
The “inverted-cup” set-up to investigate the in vitro release of ROP-ST from its oil formulation (**a**) and the release profiles of ROP-ST, ROP, and APL from the corresponding formulations into a release medium containing 0.25% SDS (**b**) (mean ± SD, *n* = 6).

In a preliminary study, the release of ROP-ST from its oil solution was investigated at 25 °C using the dialysis method [27]. Briefly, 0.5 mL of ROP-ST oil solution was placed into a dialysis bag (MWCO: 14 kDa) and immersed into 150 mL of PBS solution (pH 7.4) with 0.5% sodium dodecyl sulfate (SDS). The dialysis media were stirred with a magnetic bar at 100 r/min. An aliquot (1 mL) was withdrawn at predetermined time intervals, and ROP-ST in the samples was quantified using HPLC−UV. Surprisingly, no ROP-ST was detected until 96 h. Therefore, an “inverted-cup” method was utilized in this study to investigate the release profile of ROP-ST (Figure 3a) [28,29]. Briefly, a beaker containing 150 mL of PBS solution (pH 7.4, with 0.25% SDS) as the release medium was placed on a magnetic stirrer and stirred continuously at 100 r/min at room temperature (25 °C). A transparent glass cylinder (inner diameter: 1 cm) with two open endings was placed vertically in the beaker, with one ending immersed in the PBS solution and the other ending exposed to air. Then, 1 mL of ROP-ST oil solution was added to a glass cylinder, and the ending exposed in air was sealed with a plastic thin-film. In the meantime, the beaker was also sealed with a plastic thin-film. At predetermined time intervals, an aliquot (1 mL) of the release medium were withdrawn by a syringe, and 1 mL of fresh release medium was immediately replenished. The concentration of ROP-ST in the samples was determined by HPLC−UV, and the cumulative release of ROP-ST was calculated accordingly. The experiment was performed in triplicates.

For comparison, the release of an ROP-free base from its soybean oil solution and the release of aripiprazole lauroxil (APL) from its soybean oil solution were also investigated. To prepare the ROP oil solution, 37.5 mg of ROP-free base was dissolved and mixed in 5 mL of soybean oil. The concentration of ROP was 7.5 mg/mL (27.37 mM). To prepare the APL oil solution, 90.4 mg of APL was dissolved and mixed in 5 mL of soybean oil. The concentration of APL was 18.08 mg/mL (27.37 mM). It should be noted that all oil formulations used in the drug release study contained the same molar concentrations of the corresponding compound −27.37 mM, and the methodology to investigate the release of ROP or APL was the same as ROP-ST, as described above. Moreover, the marketed APL injectables are suspensions; therefore, the release of APL from its suspension was also studied. This was carried out by placing 5.89 mg of APL into 150 mL of PBS7.4 solution directly, which contained 0.25% SDS. The solution was also stirred and sampled in the same way as described above. APL was analyzed according to the previously reported method [30].

### 2.7. Pharmacokinetic and Tissue Retention Studies

Eighteen rats were used in the pharmacokinetic studies, which were randomly grouped (*n* = 6). ROP·HCl aqueous solution, ROP oil solution, or ROP-ST oil solution were injected into the lateral thigh muscle of the right hindlimb of the rats (7.5 mg/kg, calculated by ROP), respectively. ROP-ST oil solution (27.37 mM) was prepared as described in “Section 2.6.1”. ROP oil solution (27.37 mM) was prepared as described in “Section 2.6.4”. ROP·HCl aqueous solution was prepared by dissolving ROP·HCl powder in water with the final concentration of ROP·HCl of 8.5 mg/mL (27.37 mM). It should be noted that the administered doses of ROP·HCl aqueous solution, ROP oil solution, and ROP-ST oil solution were equal, calculated by ROP. Blood samples withdrawn from the orbital venous plexus were collected into heparinized tubes at 0, 0.25, 0.5, 1, 2, 3, 4, 6, 8, 12, 24, 36, 48, 60, and 72 h after administration. Plasma was immediately separated via centrifugation at 4000 r/min for 10 min and then stored at −20 °C.

Upon completion of the final blood sampling, the animals were then sacrificed using carbon dioxide, and the lateral thigh muscles of the right hindlimb (injection site) were excised from the animals for drug extraction and analysis. The ROP or ROP-ST that remained in the muscles was extracted by cutting the weighed samples into small pieces and soaking them in 10 mL of acetonitrile following 1 min of homogenization using an T-18 Ultra Turrax Digital Homogenizer (IKA; Staufen, Germany). The samples were filtered and then stored at −20 °C.

Before analysis, 100 μL of thawed biological sample and 50 μL of lidocaine hydrochloride methanol solution (internal standard, IS; 1600 ng/mL) were mixed and vortexed for 30 s, followed by adding 250 μL of pure acetonitrile. The mixtures were vortexed for 1.5 min and centrifuged at 12,000 r/min for 10 min, then 200 μL of supernatant was withdrawn from each sample for the quantification of ROP.

The quantification of ROP concentrations in biological samples was performed on an Acquity^®^ ultraperformance liquid chromatography system coupled with a Quattro Premier XE tandem mass spectrometer (UPLC-MS/MS, Waters Corporation, Milford, CT, USA). The chromatographic separation was achieved using an Acquity^®^ UPLC BEH C18 column (100 mm × 2.1 mm, 1.7 µm). The mobile phase consisted of 0.1% formic acid in water/acetonitrile (5/95, *v*/*v*) with a flow rate of 0.2 mL/min. The column temperature was kept at 40 °C, and the injection volume was 5 µL. For the conditions of the detector, electrospray ionization was set at a positive-ion mode (ESI+), and multiple reaction monitoring modes (MRMs) were employed for quantitative analysis with the parent–daughter ion transitions of *m*/*z* 275.2 → 126.2 for ROP, *m*/*z* 571.3 → 126.2 for ROP-ST, and *m*/*z* 235.0 → 86.0 for IS, respectively. The calibration curves were linear in the range of 3.65–365 nM for both ROP and ROP-ST, respectively, and the correlation coefficient was >0.99. The method showed good precision and accuracy for both intra- and inter-day analyses. The lower limit of quantifications (LOQ) for ROP and ROP-ST was 3.65 nM (equal to approximately 1 ng/mL of ROP). All pharmacokinetic parameters, including maximum plasma concentration (C_max_), time to reach the maximum plasma concentration (T_max_), and area under the blood concentration–time curve (AUC), were calculated using the DAS 3.0 software (BioGuider Medicinal Technology, Shanghai, China) using the non-compartmental model.

### 2.8. Pharmacodynamic Studies

Thirty rats were used in the pharmacodynamic studies, which were randomly divided into 5 groups (*n* = 6). The efficacy of pain management was investigated using an incisional rat model described previously [31,32], in which an incision on the plantar aspect of the rat’s hind paw was made after drug administration, followed by evaluations of the pain responses to mechanical stimuli. To perform the hind paw surgery, the rat was placed in a transparent acrylic box and anesthetized with 2% isoflurane. A 1 cm longitudinal incision was made through the skin of the right hind paw, beginning 0.5 cm from the end of the heel and extending toward the toes. The plantaris muscle was elevated using forceps and incised longitudinally [33]. Gentle pressure with a piece of sterilized cotton was applied for hemostasis, and the wound was sutured with two single stitches using 5-0 nylon. Upon completion of the surgery, the rat was allowed to recover from the anesthetic, which took about half an hour, and then they were administered with 200 μL of saline (model group 1), soybean oil (model group 2), ROP-HCl saline (treatment group 1), ROP oil solution (treatment group 2), or ROP-ST oil solution (treatment group 3) into the vicinity of the sciatic nerve in the right ankle, respectively. For the 3 treatment groups, the formulations used were the same as the corresponding formulation in pharmacokinetic studies; therefore, the dose administered for these groups were equal: 1.5 mg/rat, calculated by ROP-free base.

The mechanical hyperalgesia was measured by testing the paw withdrawal threshold (PWT) to mechanical stimuli using von Frey filaments (Stoelting; Wood Dale, IL, USA) [33,34]. The tests were conducted before surgery (0 h) and 1, 2, 4, 8, 24, 48, and 72 h after administration of vehicle or drug formulations. The PWT was defined as the lowest force that caused at least two withdrawals of the injured paw in three trials. Briefly, a rat was placed in custom-made individual transparent cubicles on a metal mesh floor. The filaments, with bending forces equivalent to 4, 6, 8, 10, 15, and 26 g, were introduced through the wire mesh and applied perpendicularly to the surrounding surgical area of the right paw for 3~4 s from the least to the greatest forces. The responses of the injured paw (withdrawal or not) were recorded. If there was no response to the force of 26 g, the tests were stopped to avoid further injury.

### 2.9. Statistics

Data are expressed as the mean ± SD. Outliers determined using the Dixon test were discarded. The results were evaluated statistically either by Student’s *t* test for analysis containing 2 groups or by ordinary one-way analysis of variance (ANOVA) with Tukey’s multiple comparisons test (GraphPad Prism, Version 8.0.2) for analysis containing 3 or more groups. The level of significance was determined by a *p* value: *p* > 5 × 10^−2^ was considered to indicate a nonsignificant difference, and 1 × 10^−2^ < *p* < 5 × 10^−2^ was considered to indicate a significant difference.

## 3. Results

### 3.1. Solubility and Partition Coefficient

The saturated solubilities of ROP and ROP-ST in different solutions at 37 °C are shown in Table 1. Compared to the high aqueous solubility of ROP·HCl in water, the solubility of ROP-free base in PBS 7.4 solution is only about 1 mM (~0.27 mg/mL), and the solubility of ROP-ST is even lower than the LOQ. In contrast, the ROP-free base showed moderate solubility in the soybean oil, whereas ROP-ST was miscible with the soybean oil. The extremely high oil solubility and low water solubility of ROP-ST rendered the experimental determination of its Log P value difficult. The predicted Log P value of ROP-ST was >9, demonstrating its high affinity to the oil depot.

### 3.2. Stability of ROP-ST

**Figure 4:**
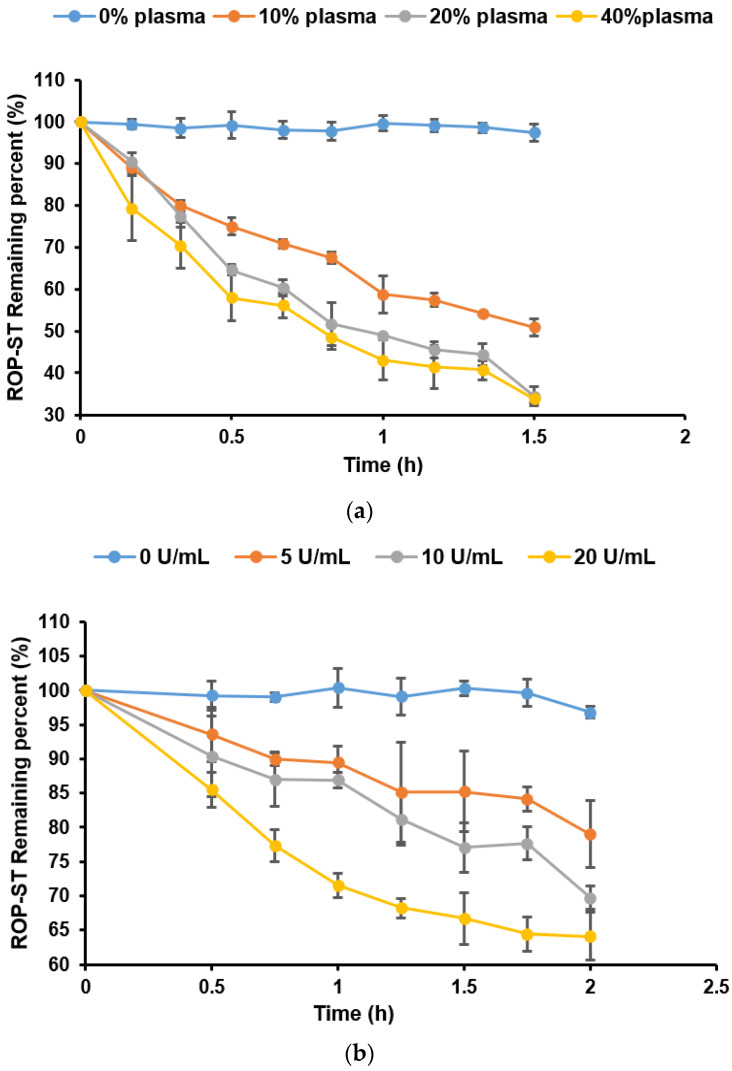
The stability of ROP-ST affected by plasma (**a**) and liver enzymes (**b**) (*n* = 3).

The enzymatic stability of ROP-ST was examined by placing the methanolic solution of the prodrug into rat plasma solution and porcine liver esterase solution. The rate of in vitro bioconversion of the ROP-ST to ROP was represented as the half-life derived from monitoring the disappearance of ROP-ST over time. The hydrolysis rate constants (*k*_obs_) of the prodrug were calculated by plotting the natural logarithm of the remaining percentage against time. The corresponding half-life (*t*_1/2_) was then calculated (*t*_1/2_ = ln2/*k*_obs_). Data at 25 °C were fitted approximately to a first-order kinetic equation for the prodrug loss (Figure 4) [26]. It should be noted that the actual hydrolytic kinetics of such lipophilic prodrugs by enzymes could be complicated in vitro and in vivo, which may not fully comply with the first-order kinetics. As shown in Table 2 and Table 3, the presence of plasma and liver esterase significantly accelerated the degradation of ROP-ST. The rate of enzymatic cleavage increases as the concentration of rat plasma or the concentration of porcine liver esterase increases. In fact, the injection site of ROP-ST is muscle, which contains less esterase than the plasma. Therefore, it is logical to hypothesize that the bioconversion rate of ROP-ST in the muscle should be slower, which enables sustained release and long-acting efficacy.

### 3.3. Evaluation of the Formulation

#### 3.3.1. Assay

The experimental results show that the ROP-ST content in the ROP-ST soybean oil solution accounts for 100.65% of the labeled amount (theoretical content).

#### 3.3.2. Stability

The results show that ROP-ST soybean oil injection has good stability at room temperature during a 6-month period: the remaining percentage of ROP-ST was 99.91%. However, the degradation became nonnegligible under high temperature (50 °C) during a 3-month period: the remaining percentage of ROP-ST was 94.50%. Therefore, ROP-ST soybean oil injection should be stored at room temperature.

#### 3.3.3. In Vitro Release

As shown in Figure 3a, the in vitro release experiments were carried out in an “inverted-cup” set-up to facilitate the release of the highly lipophilic molecules from the oil matrix. This method could diminish the effects of hindrance or adsorption of lipophilic molecules to the dialysis membrane. First of all, the sink conditions of ROP-ST, ROP, and APL were ascertained: the solubilities of ROP-ST and APL in the release media were approximately 1.5 mM and 209.3 μM, and the solubility of ROP was higher than ROP-ST; therefore, the conditions used in the release study can meet the sink conditions. As shown in Figure 3b, the release rate of ROP-ST was significantly slower than that of ROP, which is consistent with the result that ROP-ST has a higher Log P value than ROP. ROP was released almost 80% in the first 12 h, while ROP-ST maintained the sustained release for more than 72 h with a total cumulative release of 69.55%, which should be advantageous for prolonging its action in vivo. Compared with the release rates of the ROP oil solution and ROP-ST oil solution, the APL oil solution had a slower release rate, which should be attributed to the difference in the physicochemical properties of ROP-ST and APL—it is true that both ROP-ST and APL are insoluble in water, but the ROP moiety (experimental Log P value: 1.88) from ROP-ST is still more polar than the aripiprazole moiety (predicted Log P value: 4.6, data from Pubchem) from APL. Moreover, the dissolution experiment was only carried out at pH 7.4, which reflected the physiological pH value at the injection site. At this pH value, ROP moiety was ionized on its tertiary amine group due to its higher pKa value (pKa 8.07) [35], while aripiprazole, the active moiety of APL, has a lower pKa value of 7.46, making it a weak base and almost neutral in this condition. Therefore, both polarity and charge properties made ROP-ST release faster and APL from the oil base. The marketed APL injection is suspension. We found that the APL solid particles presented a higher release rate than that of APL from the oil matrix, which clearly demonstrated the effect of sustained release provided by the oil matrix to APL. APL released faster without the oil matrix, even if APL was in crystal form.

### 3.4. Pharmacokinetic Studies

**Figure 5:**
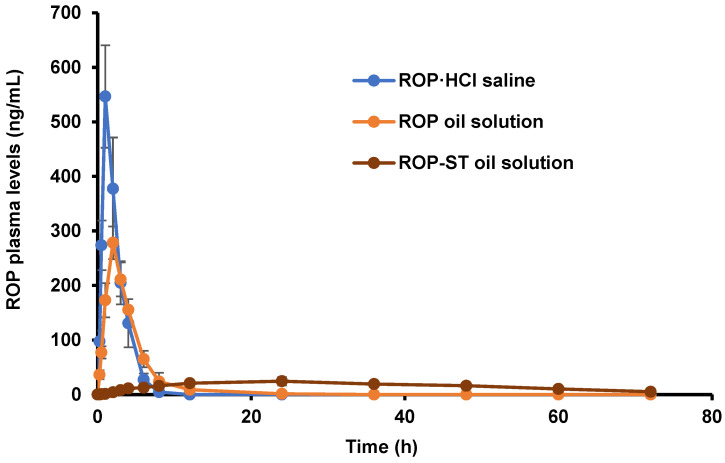
Plasma concentrations of ROP after IM administration of ROP·HCl saline, ROP oil solution, or ROP-ST oil solution in rats (mean ± SD, *n* = 6).

The plasma concentrations of ROP over time after IM injection of ROP·HCl saline, ROP oil solution, or ROP-ST oil solution, respectively, are shown in Figure 5. Their pharmacokinetic parameters are shown in Table 4. The plasma concentration of ROP in ROP·HCl saline group increased more rapidly after administration than those from the ROP oil solution group, and the peak concentration (C_max_) of the ROP·HCl saline group was nearly 2 times higher than that of the ROP oil solution group, indicating that ROP was rapidly absorbed into the blood after injection of ROP·HCl saline, whereas the slow release of ROP-free base from its oil depot reduced the input rate of the drug from the injection site to the circulation. Nevertheless, the mean residence time (MRT) of the ROP oil solution group was only about 4 h, which was not high enough. In accordance with the result of in vitro release, this could be attributed to the polarity/charged property of the ROP moiety. In this regard, encapsulation of ROP into particulate formulations would be more effective in sustaining the release of ROP locally than ROP oil formulation [16,19].

The overall plasma concentrations of ROP in the ROP-ST oil solution group were very low—the plasma concentration of ROP at 1 h was below LOQ, and the C_max_ was only 26.58 ng/mL. The T_max_ and MRT were much higher than the corresponding value of the ROP·HCl saline group and ROP oil solution group, respectively. Only a trace amount of ROP-ST can be detected before 4 h. These results suggested that ROP entered the blood very slowly because ROP-ST was released slowly from the oil base (the first rate-limiting step) and then degraded slowly into ROP by esterase (the second rate-limiting step). The in vitro stability and in vitro release data also reflected these two rate-limiting steps. The slow and sustained release of ROP in the local injection site and very limited systemic drug exposure are advantageous for long-acting local anesthesia. This result complied with the previous reports. After subcutaneous injection of a liposome-in-gel formulation of ROP into rats, it showed much smaller burst release and slower clearance than those after administration of ROP solution or ROP gel, and it released ROP at a steadier rate [17]. Similarly, when ROP was subcutaneously administered using PF-72 (a reverse thermal hydrogel), its concentration–time curve (AUC_last_) and peak concentration (C_max_) were much lower than those after administration of ROP solution [32].

The muscle tissue retention study also proved the data of the pharmacokinetic study from the other side. In this study, no ROP can be detected in the lateral thigh muscle of the right hindlimb after injectable administration of ROP·HCl saline or ROP oil solution for 72 h. In contrast, 52.7 ng/g of ROP and 116.4 μg/g of ROP-ST can be found in the muscle tissue after injectable administration of ROP-ST oil solution for 72 h, which unequivocally showed the sustained-release property of ROP-ST oil formulation in local tissue.

### 3.5. Pharmacodynamic Studies

**Figure 6:**
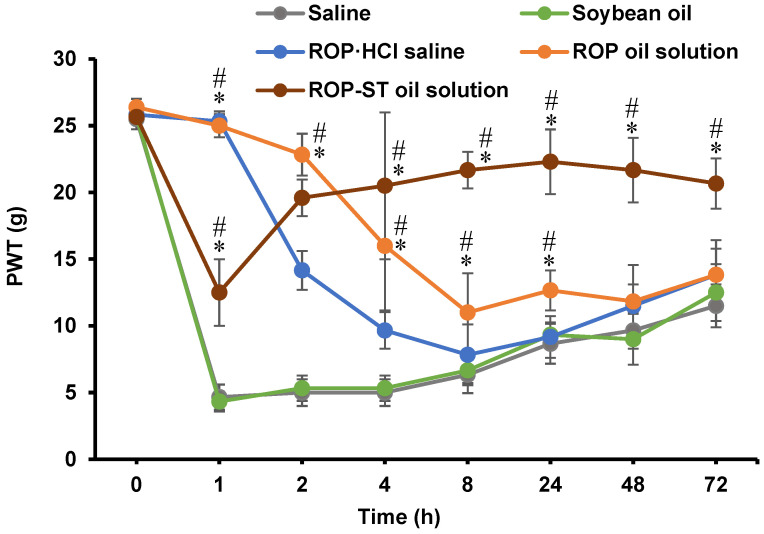
Paw withdrawal threshold (PWT) after administration of 200 μL of saline, soybean oil, ROP·HCl saline, ROP oil solution, or ROP-ST oil solution into the vicinity of the sciatic nerve in the right ankle of the rats using the von Frey test (mean ± SD, *n* = 6). * *p* < 0.05, compared with the saline group; # *p* < 0.05, compared with the ROP-HCl saline group.

The results of the Von Frey test are shown in Figure 6. The PWT is the minimum amount of force required to cause a rodent to withdraw its hind paw from a stimulus. It is a measure of mechanical nociception, or the ability of an animal to detect harmful stimuli. The PWT is typically measured using the von Frey filament test, which involves applying calibrated plastic filaments to the bottom of a rodent’s hind paw. The filaments are applied in increasing order of stiffness until the animal withdraws its paw [36]. The PWT values of the rats in the saline and soybean oil groups were not different from those of the treatment groups before surgery (i.e., 0 h) but were significantly lower than those of the treatment groups after surgery, indicating that surgery-induced hyperalgesia was established. PWT values of the ROP·HCl saline group and ROP oil solution group were the highest at 1 h, but the former one decreased more rapidly from 2 h to 8 h than the latter one: the PWT value of the ROP·HCl saline group was already not significantly different from that of the two model groups at 8 h, but the value of the ROP oil solution group was still significantly higher than that of the model groups at 24 h, which suggested that ROP oil solution apparently showed some slow-release properties in vivo. Although the PWT value of the ROP-ST oil solution group was significantly higher than that of the model groups at 1 h, it was also significantly lower than those of the ROP·HCl saline group and ROP oil solution group. Moreover, the PWT values of the ROP-ST oil solution group were not only significantly higher than those of the model groups but also significantly higher than those of the ROP oil solution group from 8 h to the end of the experiment. The results should be attributed to the fact that after the ROP-ST oil solution was injected into the vicinity of the sciatic nerve in the right ankle, it took some time for ROP to release and reach the nerves: both the slow release of ROP-ST from the oil depot and the hydrolysis of ROP-ST to ROP by tissue esterase contributed to the long-acting analgesia. In addition, it should be noted that the ROP-ST oil solution group still showed a certain analgesic effect at 72 h, which may imply that the duration of the analgesic effect could be more than 72 h. This needs to be further verified.

The values of PWT can correspond to the results of pharmacokinetics: after IM injection of ROP·HCl saline, the plasma concentration of ROP reached the peak within a short time and then decreased rapidly, indicating that ROP·HCl was quickly absorbed into the blood rather than detained in the tissue. This explains that ROP·HCl saline could only maintain effective local analgesia within 4 h after administration. In contrast, the very low overall plasma concentration of ROP after administration of ROP-ST oil solution suggested that the slow and sustained release of ROP was ongoing for a long period in the injection site, which in turn translated to a long period of ROP exposure around the sciatic nerve. The data on muscle tissue retention directly proved this phenomenon.

It should also be noted that although the ROP-ST oil solution was able to provide effective analgesia for a longer duration, the analgesic effect was apparently weaker than that of the ROP·HCl saline and ROP oil solution groups within 1~2 h after administration (i.e., the very early stage of after injection). It implies that the use of ROP-ST oil solution alone as an analgesic agent to manage postoperative pain is inadequate. The addition of ROP-free base into the ROP-ST oil solution should theoretically solve this problem—the release of ROP from the oil solution is responsible for the fast onset of analgesia, whereas the release of ROP after hydrolysis of ROP-ST can provide long-period analgesia. The ratio and the amounts of ROP-free base and ROP-ST added into the oil depot will be tailored more precisely in future studies. Given the fact that the excipient used to formulate ROP-ST oil injection was soybean oil, which is a commonly used excipient that is injected into the muscle, and the hydrolyzed product of ROP-ST, i.e., stearic acid, also participates in the synthesis of lipid molecules such as triglycerides in the body; therefore, it is unlikely to induce significant inflammatory irritation after injection. We also did not observe any irritation effects in or around the injection site. However, the toxicity of the formulation still needs to be investigated in future studies.

## 4. Conclusions

The maintenance of effective local analgesia in a relatively long period is essential to manage acute postoperative pain. The marketed injectable formulations can achieve effective local analgesia in 3 days by releasing local analgesics slowly from sophisticatedly designed and manufactured liposomal systems or in situ gelling systems. In this study, a simple strategy combining prodrug modification and an oil-based depot was also proved effective. Inspired by many successful examples using prodrug strategies to prolong the half-life of drugs, a novel *N*-acyloxymethyl prodrug of ROP, i.e., ROP-ST, was synthesized and formulated into an oil-based depot formulation. The formulation showed appreciably slow release in vitro and low peak concentration of ROP in the plasma after intramuscular administration of ROP-ST oil formulation in rats. Injection of ROP-ST oil solution provided late onset but appreciably long analgesia in rats. Moreover, given that the ROP-ST oil solution group still showed a certain analgesic effect at 72 h, it may imply that the analgesic effect could exist for more than 3 days. These findings suggest that ROP-ST oil formulation offers a promising new approach to managing acute postoperative pain with low systemic exposure and long periods of local analgesia.

## Figures and Tables

**Table 1:** The saturated solubility and Log P of ROP and ROP-ST (mean ± SD, *n* = 3).

	M.W. ^a^	S_PBS 7.4_ ^b^	S_oil_^c^	Log P_exp._ ^d^	Lop P_pred._ ^e^
ROP	274.40	1.04 ± 0.04	37.05 ± 0.53	1.88 ± 0.17	2.78
ROP-ST	570.48	<LOQ	Miscible	Not available	9.16

^a^ M.W., molecular weight. ^b^ S_PBS 7.4_, the solubility of ROP and ROP-ST in PBS 7.4 solution, mM. ^c^ S_oil_, the solubility of ROP and ROP-ST in soybean oil, mM. ^d^ Log P_exp._, experimental Log P. ^e^ Log P_pred._, predicted Log P.

**Table 2:** First-order hydrolysis rate constants *k* (h^−1^) and *t*_1/2_ (h) of ROP-ST in plasma solutions (25 °C, mean ± SD, *n* = 3).

Concentration of Rat Plasma (*v*/*v*)	0% ^a^	10%	20%	40%
*k* × 10^3^ (h^−1^)	9.1 ± 3.2	436.6 ± 17.2	665.0 ± 35.2	666.9 ± 30.7
*t*_1/2_ (h)	82.25 ± 26.18	1.59 ± 0.06	1.04 ± 0.06	1.04 ± 0.05

^a^ 0%, saline solution without rat plasma.

**Table 3:** First-order hydrolysis rate constants k (h^−1^) and *t*_1/2_ (h) of ROP-ST in liver esterase solutions (25 °C, mean ± SD, *n* = 3).

Concentration of Porcine Liver Esterase (U/mL)	0 ^a^	5	10	20
*k* × 10^3^ (h^−1^)	8.0 ±1.3	107.9 ± 29.0	159.6 ±1.3	226.1 ± 24.7
*t*_1/2_ (h)	88.43 ± 14.94	6.78 ± 1.99	4.34 ± 0.03	3.09 ± 0.36

^a^ 0 U/mL, phosphate buffer (pH 8.0) without liver esterase.

**Table 4:** Pharmacokinetic parameters after IM administration in rats (*n* = 6).

Formulations	AUC_(0–72 h)_ (μg/L·h)	MRT _(0–72 h)_ (h)	T_1/2_ (h)	T_max_ (h)	C_max_ (μg/L)
ROP·HCl saline	1383.87 ± 220.79	2.19 ± 0.24	1.36 ± 0.30	1.17 ± 0.41	568.98 ± 78.80
ROP oil solution	1181.14 ± 259.29	4.02 ± 0.69 *	2.17 ± 0.44 *	2.0 ± 0.00 *	278.41 ± 32.83 *
ROP-ST oil solution	1145.28 ± 125.28	32.38 ± 4.35 ^#^	17.80 ± 8.38 ^#^	28.00 ± 12.40 ^#^	26.58 ± 6.94 ^#^

* *p* < 0.05, compared with ROP·HCl saline; ^#^ *p* < 0.05, compared with ROP·HCl saline.

## Data Availability

Data are contained within the article.

## Supplementary Materials

The following supporting information can be downloaded at: https://www.mdpi.com/article/10.3390/pharmaceutics17010037/s1.
