# Supplementary material for: An Injectable Oil-Based Depot Formulation of N-Acyloxymethyl Prodrug of Ropivacaine for Long-Acting Local Analgesia: Formulation Development and In Vitro/In Vivo Evaluation
Source: Pharmaceutics. 2024 Dec 30;17(1):37. doi: 10.3390/pharmaceutics17010037 (PMC11768344; doi:10.3390/pharmaceutics17010037)

SUPPLEMENTARY MATERIAL

An Injectable Oil-based Depot Formulation Of *N*-acyloxymethyl Prodrug Of Ropivacaine For Long-acting Local Analgesia: Formulation Development And In vitro/In vivo Evaluation

Xiaowei Liu [1, $, †], Ruihan Zhao [2, †], Peijie Xu [1], Jianqiang Qian [1], Peiyan Zhang [1, 3], Xudong Xie [1], Yong Ling [1], Qimin Ge [1, 4, *], Yong Chen [1, **]

[1] School of Pharmacy, Nantong University, 9 Seyuan Road, Nantong 226019, Jiangsu Province, China;

[2] School of Pharmacy, China Pharmaceutical University, 639 Longmian Avenue, Nanjing 211198, Jiangsu Province, China;

[3] Jiangsu Provincial Institute of Materia Medica, 26 Majia Street, Nanjing 211816, Jiangsu Province, China;

[4] Department of Pharmacy, The First People's Hospital of Yancheng, 66 Renmin South Road, Yancheng 224006, Jiangsu Province, China;

[$] Current address: Department of Pharmacy, Yancheng TCM Hospital Affiliated to Nanjing University of Chinese Medicine, 53 Renmin Middle Road, Yancheng 224000, China;

[*] Correspondence: scuchen2003@ntu.edu.com (Y.C.); gqm2022gqm@163.com (Q.G.); Tel.: +86-189-1161-7152 (Y.C.); +86-183-5128-3029 (Q.G.)

† These authors contributed equally to the work.

SUPPLEMENTARY MATERIAL

1. Characterization of the chemical structure of ROP-ST

ROP and ROP-ST were placed in NMR tubes. The substances were dissolved in $CDCl_3$ and allowed to stand within the NMR spectrometer chamber. The ^{1}H-NMR spectra were recorded using an AVANCE III HD 400 instrument (Bruker Corporation, Massachusetts, United States) Subsequently, high-resolution mass spectra were determined and documented utilizing the API QSTAR® Pulsar high-resolution mass spectrometer (Applied Biosystems, Inc.) to ascertain the molecular formula of the synthesized compounds. The ^{1}H NMR spectrum of ROP and ROP-ST were shown in Fig. S1, and the mass spectrum of ROP-ST were shown in Fig. S2, respectively.

ROP-ST: ^{1}H NMR (400 MHz, $CDCl_3$) δ: 0.88 (t, J = 3.7 Hz, 6 H, $2CH_3$), 1.69–1.42 (m, 16 H, $8CH_2$), 2.01 (m, 5 H, CH_3, CH_2), 2.19 (t, J = 6.0 Hz, 6 H, $3CH_2$), 2.34 (m, 8 H, $4CH_2$), 2.63 (m, 2 H, CH_2), 3.24 ~ 3.14 (m, 2 H), 3.46 (dd, J = 9.6, 3.3 Hz, 1 H), 5.43 (m, 1 H, CH), 5.80 (dd, J = 19.0, 10.4 Hz, 2 H, CH_2), 7.14 (m, 2 H, 2ArH), 7.19 (dd, J = 8.5, 6.4 Hz, 1 H, ArH). HRESI-MS ($C_{36}H_{62}N_2O_3$ + H): Calc. 571.4839; Exp. 571.4826.

2. Validation of HPLC-UV analytical methods

2.1 Quantification method

Quantification of ROP-ST was performed using a HPLC−UV system consisted of a LC-10AT VP pump, a SPD-10A VP UV–vis detector, a CTO-10A column oven, a SIL-10AF auto sampler and a SCL-10A VP controller (Shimadzu Corporation; Kyoto, Japan). Data were collected and processed using LC-solution software. Isocratic elution was performed on a Diamonsil C18 column (150 mm × 4.6 mm I.D., 5 μm), and an EasyGuard C18 guard column (10 × 4.0 mm I.D., 5 μm) was mounted upstream from the analytical column (Dikma Technologies; Beijing, China). The column temperature was kept at 35 °C, and the UV absorbance wavelength was set at 215 nm. The mobile phase, comprising 88% (*v/v*) acetonitrile and 12% (*v/v*) water with 0.1% (*v/v*) trifluoroacetic acid, was used after filtering through a filter membrane (pore size: 0.45 μm) and degassing within an ultrasonic water bath (KQ-500VDE Ultrasonic Cleaner;

Jiangsu, China) for 40 min. The flow rate was maintained at 1.0 mL/min, and the injection volume was 20 μL. The representative chromatograms were shown in Fig. S3.

2.2 Linearity and the lowest limit of quantification

The stock solution of ROP-ST (1000 μM) was prepared by dissolving 5.70 mg ROP-ST in 10 mL methanol in the aid of ultrasonication, which was then diluted by methanol to obtain a series of calibration standards. The calibration curves of ROP-ST were constructed by plotting the the peak area of ROP-ST against the concentration of the corresponding analyte (200, 150, 100, 75, 50, 20, 10, 5, 2 and 1 μM). The linearity was evaluated using the calibration curve. The lowest concentration within the calibration standards at which the signal-to-noise ratio was >10 with an acceptable accuracy (within ±20%) and precision (relative standard deviation, RSD, <20%), was defined as the lowest limit of quantification (LLOQ).

As shown in Fig. 4S, the calibration curve was linear in the range of 1–200 μM, and the correlation coefficient was > 0.9998. The lower limit of quantification (LOQ) of ROP-ST was 1 μM (equal to 0.57 μg/mL).

2.3 Precision and Accuracy

The methods were validated with 3 replicates at 10, 100 and 200 μM and showed good intra-day precision and accuracy (Table S1).

SUPPLEMENTARY MATERIAL

Tab. S1 Precision and accuracy of the analytical method used to quantify ROP-ST (n = 3)

	$C_{theo.}$[a] (μM)	$C_{exp.}$[b] (μM, mean ± SD)	Precision (%)[c]	Accuracy (%)[d]
Intra-day	10	10.11 ± 0.04	0.40	101.10
	100	99.00 ± 0.54	0.55	99.00
	200	199.20 ± 2.43	1.22	99.60
Inter-day	10	10.16 ± 0.06	0.59	101.56
	100	99.57 ± 0.30	0.30	99.56
	200	199.59 ± 0.66	0.33	99.80

[a]$C_{theo.}$: theoretical concentration, μM.

[b]$C_{exp.}$: experimental concentration, μM.

[c]Precision (%) = (SD/mean) ×100%.

[d]Accuracy (%) = (experimental concentration/theoretical concentration) ×100%.

SUPPLEMENTARY MATERIAL

FIG. LEGENDS

Fig. S1. ^{1}H NMR spectrum of ROP (a) and ROP-ST (b)

Fig. S2. High resolution mass spectrum of ROP (a) and ROP-ST (b)

Fig. S3. Representative chromatograms of HPLC to quantify ROP-ST. ROP-ST solution (a), Blank soybean oil solution (b), Blank rat plasma (c)

Fig. S4. HPLC standard curve of ROP-ST

SUPPLEMENTARY MATERIAL

Fig. S1.

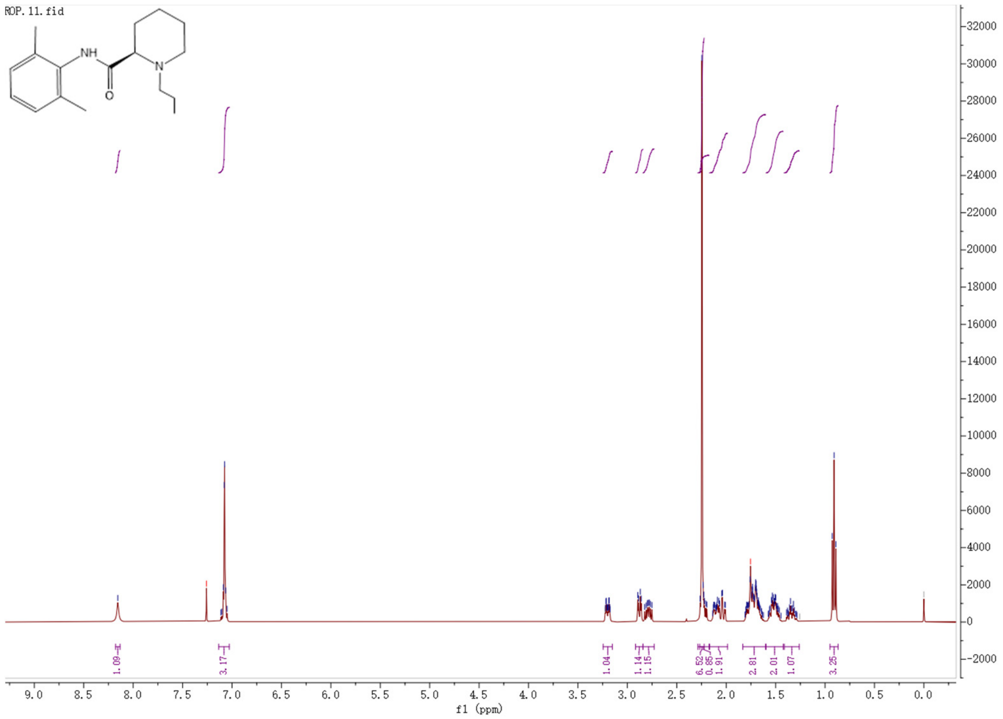

(a)

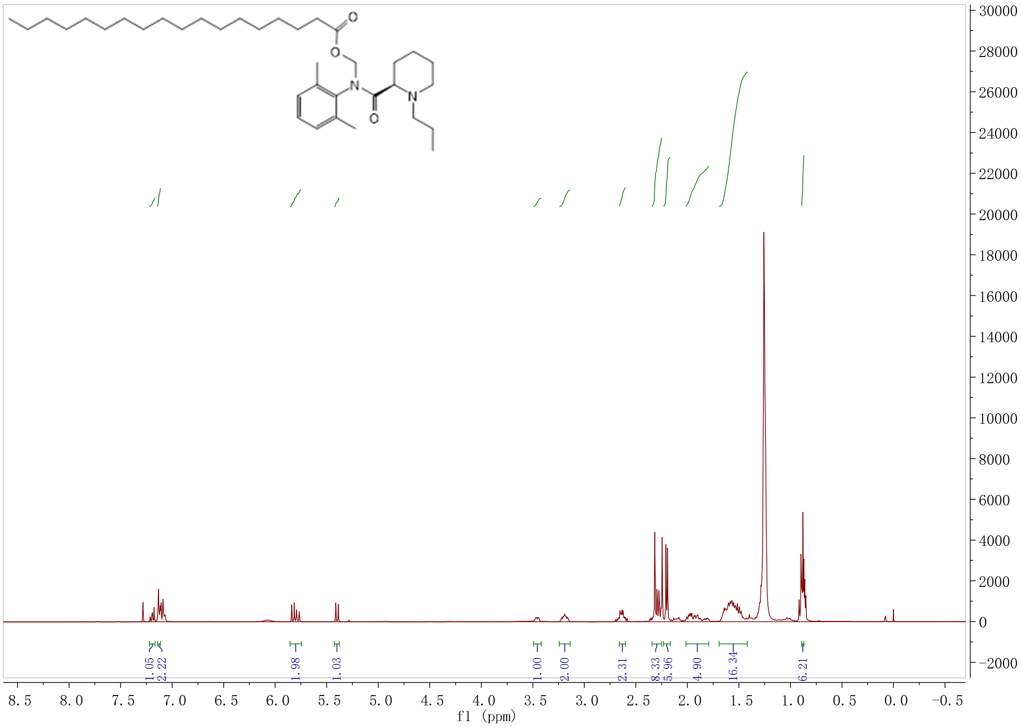

(b)

SUPPLEMENTARY MATERIAL

Fig. S2.

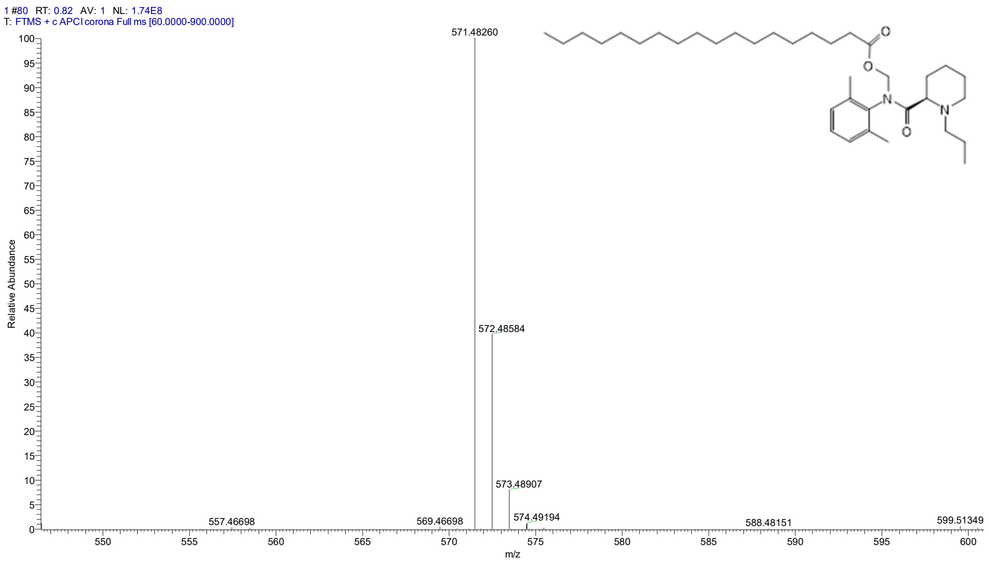

SUPPLEMENTARY MATERIAL

Fig. S3

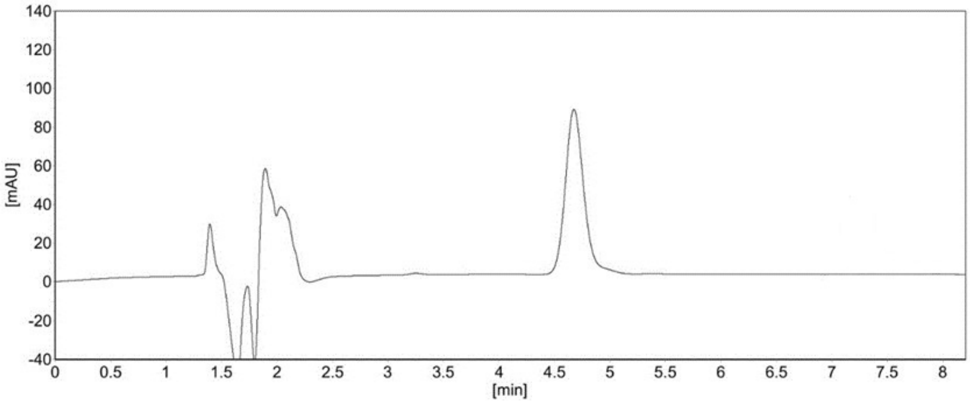

(a)

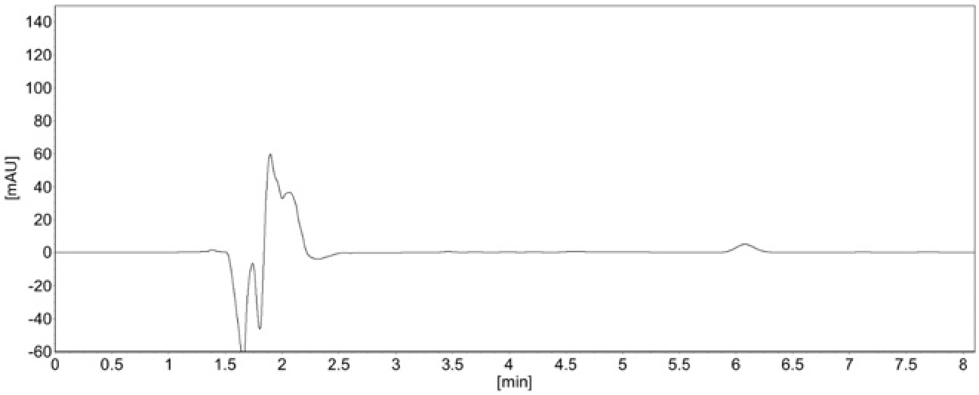

(b)

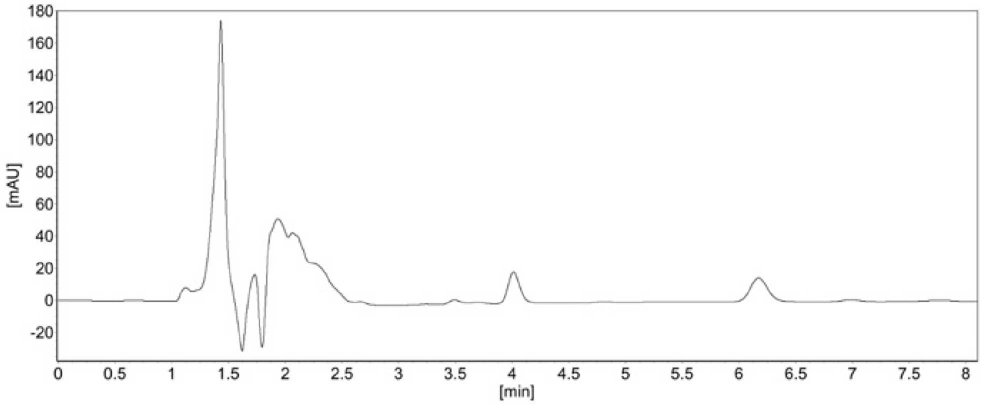

(c)

SUPPLEMENTARY MATERIAL

Fig. S4.

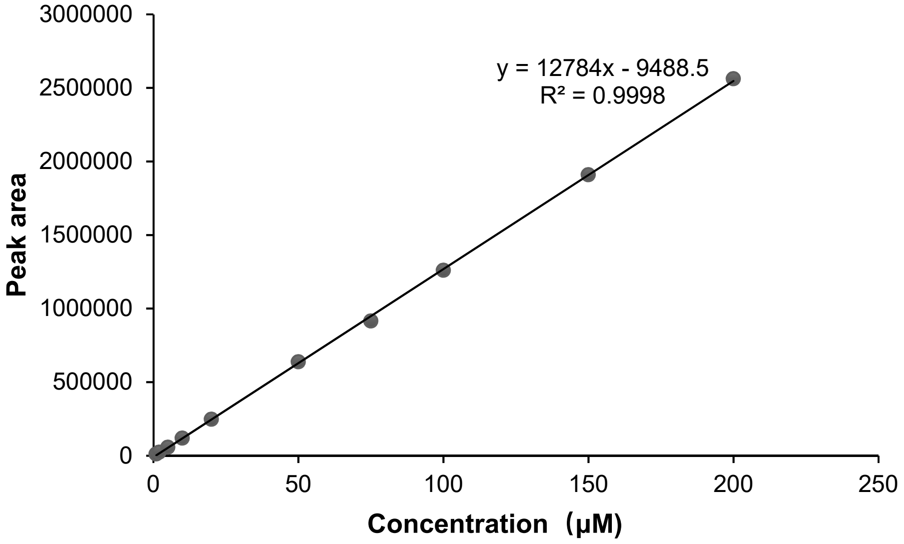

Supplement: Supplementary file 1 [file pharmaceutics-17-00037-s001.zip › pharmaceutics-3303816-supplementary.pdf]
